# Salivary retention of silver diamine fluoride

**DOI:** 10.6026/97320630018420

**Published:** 2022-04-30

**Authors:** Zohra Jabin, V Vishnu Priya, Iffat Nasim

**Affiliations:** 1

**Keywords:** Silver Diamine Fluoride, fluoride retention, enamel demineralization, primary teeth, saliva

## Abstract

The topically applied fluorides are efficacious in both prevention against caries attack and inhibition of virulent bacteria. The purpose of this clinical trial was to assess the fluoride concentration in saliva before and after 38% SDF, 5% NaF and 1.23% ApF gel application on enamel and duration of its availability at different time intervals. The present randomized clinical trial was conducted among 60 healthy children aged between 6-12 years where at baseline the participants were instructed to spit for 2 min in sterile containers and the first saliva sample (S1) was taken. The participants were then randomly allocated into 3 different groups in which 38% Silver diamine fluoride, 5% Sodium fluoride and 1.23% ApF gel were applied respectively. The second saliva sample (S2) was collected after 5 min and patients were called after 1 hour for third saliva sample collection. The fluoride concentration was measured in the salivary samples. ANOVA test was used for evaluation and chi square t test was conducted for comparison of 3 groups. The fluoride concentration is comparatively slightly higher for the group receiving SDF than NaF and ApF at baseline, 5 min and 1 hour time interval but is not statistically significant. The mean scores of Fluoride concentration of the three groups were statistically significant at 5 min (F=63.556, p<0.0005) and 1 hour time interval (F=17.577, p<0.0005). Slightly increased salivary fluoride retention was observed post SDF application at 5min and 1 hour time interval when compared to Na F and ApF gel application. The present trial also concluded that topical fluoride application increases fluoride bioavailability in saliva thereby increasing tooth remineralization.

## Background:

There is a strong evidence and literature support regarding the clinical effectiveness of topically applied fluorides. They are efficacious in both prevention against caries attack and inhibition of virulent bacteria [1-3]. These actions of topical fluorides are largely attributed to their constant presence in the oral environment.The sustain release of fluoride present in low concentration in saliva and plaque exerts protective action against bacteria [4]. Incorporation of gelling agent, sodium carboxy-methyl cellulose in APF provides viscosity, thus it can be applied by using custom made trays. On application of pressure, this gel mimics a solution that facilitates gel penetration [5]. It is suggested that fluoride ion is directly toxic to virulent oral bacteria like S mutans when topical fluorides are applied in high concentrations. And this Suppression may last for extended period of time. SDF is a novel topical fluoride solution with high concentration of fluoride (44,800) ppm. Silver diamine fluoride (SDF) is an alkaline, colourless, topical fluoride solution. The two main components, fluoride and silver, along with ammonia forms a highly stable silver halide complex. The role of silver is to inhibit the bacterial action while fluoride aids in remineralizations of tooth. Ammonia dramatically stabilizes the high concentrations of silver and fluoride and increases salivary retention of SDF, thereby prolonging its action [6]. Therefore, it is of interest to assess the fluoride concentration in saliva and plaque at different time intervals following professional application of 38% SDF,1.23 % Apf and 5% NaF.

## Methodology:

### Study design:

The present study was a randomised double blind clinical trial which was conducted among sixty healthy children aged between 6-12 years. The present trial was done in accordance with the CONSORT guidelines (Figure 1 - see PDF).Ethical approval was obtained from the institutional ethical committee (STP/SDMDS2015PED42D).

### Eligibility criteria:

Patients were examined and certain inclusion and exclusion criteria were employed for the selection of study subjects.

### Inclusion criteria:

1. Subjects with sound non carious teeth

2. Subjects free of any soft or hard tissue pathologic condition.

3. Subjects with normal salivary flow rate (0.20 - 0.9 ml/min)

### Exclusion criteria:

1. Subjects with non- cavitated/ cavitated dental lesions

2. Subjects with medical, physical or mental disability

3. Subjects who took antibiotics within one month of commencement of the study.

4. Subjects who underwent fluoride application in last 3 months.

### Baseline Information:

Complete details of child’s personal and medical history were obtained before enrolling them for the study. History regarding their recent medication and treatments were taken along with their diet preferences. The participants were clinically examined with the help of a mouth mirror and an explorer. A written informed consent forms was filled by parents or guardian of the subjects who participated in the study. Oral hygiene instructions, tooth brushing demonstrations and diet counselling, were given to children during their visit for the study.

### Sample size determination:

The minimum sample size recommended for each group (SDF, APF, NAF) was 17 considering a study power of 80% (1-β), to detect statistical significance of 5% (α= 0.05) and effect size (f) 0.39 using G Power 3.1.Overall recommended sample size according to estimation was 65 which were rounded off to 60 to have a minimum sample of 20 in each group. Initially a total number of 105 patients were screened. 60 children that fulfilled the inclusion criteria and those with written parent consent were included in the study. The selected study participants were randomly assigned to 1 of 3 groups (20 children per group):

1. Group I - SDF(38% Silver diamine fluoride (Fagamin ™)

2. Group II - APF gel (1.23% Pascal ™)

3. Group III - NaF-5% Sodium fluoride Varnish (VOCO ™)

### Randomization & Blinding:

The present study was a double blind trial where the subjects and the investigator was blinded. To prevent bias a trained clinical assistant, was not involved in the study conducted computer generated randomization.

### Saliva sample collection:

On the day of saliva sample collection participants were instructed to have an early morning breakfast and report to the department in morning hours. This was ensured to maintain a specified gap of 90 minutes prior to collection of samples and to avoid any influence of food consumption on the salivary composition along with minimizing the effect of diurnal variation. Saliva samples were collected from each participant at three intervals i.e baseline, 5 min and 1 hr post fluoride application. For baseline sample collection each subject was given a plastic container and asked to evacuate at least 2 ml of un stimulated saliva for more than five minutes into the container.

After collection of baseline samples, oral prophylaxis was carried out and application of three different fluoride agents was done. Oral cavity was isolated with cotton rolls & saliva ejector and teeth were dried with gentle blow of air for 30s using three way air syringe. Applicator tips were used for the application of SDF and NaF on the teeth for a period of 4 min, after which the teeth were cleaned with gauze to prevent the swallowing of any residual topical agent. For APF application, fluoride tray was loaded upto one-third level and placed in the oral cavity for several minutes, while saliva ejector was in place for clearance of any residual fluoride in the oral cavity. The next samples were collected at duration of 5 min and an hour post fluoride application.

### Fluoride estimation:

Sample numbers were labeled on the saliva and they were stored at a temperature below 4oC.Later they were transferred to the lab. The concentration of fluoride in saliva was determined with a fluoride-selective electrode. The fluoride concentration was measured in the salivary samples which were collected at three time intervals that is at baseline (S1), 5 min (S2) and 1 hour (S3) after application.

### Statistical analysis:

The values of fluoride levels were obtained from lab and were tabulated accordingly. This numerical data were later subjected for statistical analysis using SPSS software. The mean values and ANOVA test were used for evaluation of the data.

## Results:

The present clinical was undertaken to know the salivary fluoride concentration of the participants who reported to the Department of Pedodontics and preventive dentistry. 60 study participants were recruited in the study and were randomly assigned into 3 different groups each comprising of 20 participants. Table 1(see PDF) describes the mean values of salivary fluoride concentration of three groups at three time intervals that is at the baseline (S1) prior to the application of fluorides and post application at 5 min (S2) and 1 hour (S3) time interval. The fluoride concentration is comparatively slightly higher for the group receiving SDF than NaF and ApF at baseline, 5 min and 1 hour time interval but is not statistically significant. Table 2(see PDF) describes the intra group comparison within three groups of mean fluoride concentration at different time intervals that is baseline, 5 min and 1 hour interval. When using an ANOVA with repeated measures with a Greenhouse-Geisser correction, the mean scores of Fluoride concentration of the three groups were statistically significant at 5 min (F=63.556, p<0.0005) and 1 hour time interval (F=17.577, p<0.0005)

## Discussion:

Since the discovery of its anti-cariogenic potential, fluorides have been considered the forefront of preventive dentistry. SDF has been widely used as anti- hypersensitivity agent and for caries arrest for over two decades. The present study was conducted to evaluate F levels in saliva, after treatment with topical F agents intended for professional use. The retention of F in the mouth after topical fluoride treatment is considered to be an important factor in the clinical efficacy of Fluoride which is dependent on its concentration and method of delivery. Although home use fluoride agents like rinses and toothpastes, provide higher fluoride concentrations, Zero D T et al suggested that these methods did not appear to make a clinically significant contribution to Fl levels in the mouth [7]. Professionally applied topical F products are available in a wide variety having different concentrations of fluoride. Effectiveness of fluoride varnishes in the prevention of dental caries has been widely documented in the literature [8-10]. They form a coat on the tooth surface thereby providing a fluoride in a highly concentrated form. Also, varnish holds fluoride over the tooth surface for a longer duration than other fluoride products. It shows benefits even when it is applied to the dried teeth without prior prophylaxis [11]. Topical fluoride products balance de-mineralisation and remineralization cycle in saliva by enhancing fluoride uptake during demineralization and boost re-mineralization. In this process saliva act as fluoride reservoir and exert both topical and systemic effect [12,13]. SDF is a novel agent material that is effective in increasing enamel remineralization due to its high fluoride content. It is available at affordable cost, offers easy manipulation with non-invasive application and does not require elaborate armamentarium [14-16]. The constant presence of fluoride in saliva aids in replenishing the net mineral lost from tooth surface due to pH changes in oral cavity [16]. This presence can be ensured by using SDF, which is a novel fluoride agent. The time effect plays an important role in oral bioavailability of fluoride. The presence of ammonia in SDF stabilizes the fluoride in solution for extended periods and increases its bioavailability. The present study results were in accordance with this fact and in our study the levels remained elevated up to five hours. Although SDF has remarkable benefits when used as a preventive agent, however it does have minor side effects like the staining of lesions.. The color change where silver tarnishes to black is an index of the effectiveness of the treatment, it indicates success and all lesions that are completely black are apparently arrested.

## Conclusion:

The present trial concluded that topical fluoride application increases fluoride bioavailability in saliva thereby increasing the re-mineralization of teeth. Increased salivary fluoride retention was observed post SDF application at 5 min and 1 hour time interval when compared to Na F and ApF gel application. The use of SDF and its applications in vivid aspects of dentistry should be explored with further research in this field.
